# MEA-Based Graph Deviation Network for Early Autism Syndrome Signatures in Human Forebrain Organoids

**DOI:** 10.34133/cbsystems.0441

**Published:** 2025-11-06

**Authors:** Arianna Mencattini, Giorgia Curci, Alessia Riccardi, Paola Casti, Michele D’Orazio, Joanna Filippi, Gianni Antonelli, Erica Debbi, Elena Daprati, Wendiao Zhang, Qingtuan Meng, Eugenio Martinelli

**Affiliations:** ^1^ Department of Electronic Engineering, University of Rome Tor Vergata, 00133 Rome, Italy.; ^2^ Interdisciplinary Center for Advanced Studies on Lab-on-Chip and Organ-on-Chip Applications (ICLOC), 00133 Rome, Italy.; ^3^ Department of System Medicine, University of Rome Tor Vergata, 00133 Rome, Italy.; ^4^ The First Affiliated Hospital, Multi-Omics Research Center for Brain Disorders and Department of Neurology, Hengyang Medical School, University of South China, Hunan 421001, China.; ^5^ The First Affiliated Hospital, Clinical Research Center for Immune-Related Encephalopathy of Hunan Province, Hengyang Medical School, University of South China, Hunan 421001, China.

## Abstract

Multi-electrode arrays (MEAs) are a key enabling technology in the development of cybernetic systems, as they provide a means to understand and control the activity of neural populations linking brain microtissue dynamics with electronic systems. MEAs allow high-resolution, noninvasive recordings of neuronal activity, creating a powerful interface for investigating in vitro brain development and dysfunction. In this work, we introduce a novel deep learning framework based on a graph deviation network (GDN) to analyze spiking activity from human forebrain organoids (hFOs) and predict network-level alterations associated with autism spectrum disorder (ASD) risk. Our method extends traditional spike and burst analysis by encoding amplitude-modulated spike trains as dynamic graphs, enabling the extraction of meaningful topological descriptors. These graph-based features are then processed to detect deviations in network organization induced by neurodevelopmental perturbations. As proof of concept, we examine the impact of valproic acid (VPA), a known environmental ASD risk factor. VPA disrupts synaptic signaling in hFOs, reducing efficiency, increasing path length, and decreasing input connectivity. Despite biological variability, the GDN consistently detects early dysfunction within 24 h post-exposure and captures transient millisecond-level events. This supports MEA-coupled hFOs as predictive platforms for ASD risk assessment and real-time neurotoxicity screening.

## Introduction

The introduction of multi-electrode arrays (MEAs) has marked an important breakthrough in the ability to study in vitro models of the human brain and its complex functioning in both healthy and disease states [1,2]. This technology enables noninvasive recordings of neuronal signals, thus providing additional information to more conventional electrophysiological techniques such as patch clamping [3,4] and calcium imaging [5]. Compared to those approaches, MEA utilizes electrodes to enable simultaneous electrical activity measurement of neuronal networks over long time periods, potentially months. Because of the extremely high temporal resolution, in the sub-millisecond scale, and the noninvasiveness, MEA analysis is very powerful for the identification of synaptic activity and its subtle pathological dysfunctions. MEA systems are now used to record extracellular action potentials from neuronal cells cultured directly on top of the recording electrodes. Simultaneous recording of spiking activity from multiple spatial locations within a culture allows inspection of population-wide firing activity and understanding of how network dynamics may be altered in disease states.

Standardly, MEA signals are processed to extract firing and bursting rates [6], which allow for a quantitative assessment of the joint electrical activity across the contacted areas of the brain model [7], revealing specific behavior at the single spike level [8]. In Ref. [9], the authors measured a small subthreshold sodium current due to small synaptic depolarizations that elapse in the inter-spike interval. In Ref. [8], by using a 32-channel MEA, the authors classified extracellular spikes waveforms, observed in primary wallaby visual cortex, into 5 distinct typologies: regular, fast, triphasic, compound, and positive, each with a different origin. The study demonstrated that the bursting and spiking rates can vary importantly across different waveforms, highlighting the importance of conducting more detailed time-dependent analyses of both single- and inter-electrode spike activity. Recently, attempts have been made to optimize spike sorting ability [10], allowing a finer discrimination of spike waveforms in models of Alzheimer’s disease. To date, compared with existing benchmark strategies for multichannel spike sorters [11–13], PseudoSorter [10] exhibits good performances despite not incorporating targeted approaches like drift correction or template matching. When applied to human pluripotent stem cells (hPSCs) using low-density MEA equipment [14], existing approaches encounter several challenges related to the low spatial resolution of each electrode and the difficulty of embedding a multichannel recording analysis. As widely demonstrated [7], multichannel analysis of spike recordings provides crucial information regarding the synaptic activity of the tissue recorded (brain, neural culture, organoid, etc.), especially when applied to hPSCs. A few attempts have been made to extend the single-spike waveform analysis to the multichannel recordings’ dataset [15]. In this context, interesting approaches framed the spike sorting and classification problems within deep neural networks [16]. By exploiting supervised approaches [17] based on convolutional neural networks (CNNs) [18], generative adversarial networks [19], multi-layer perceptron [20], or recurrent neural networks, especially long short-term memory networks [21], several authors proposed various methods to automatically detect and/or sort spikes in MEA datasets—although these methods may fail to detect subtle variations in the activity pattern.

Despite the extensive literature on spike analysis, few studies have leveraged neural network architectures capable of uncovering explainable network-level rules beyond traditional spike counting or sorting [22–24]. MEA recordings generate data as rich multivariate time series that encode the dynamic interactions across neuronal populations. However, conventional analyses often rely on averaging signals, which can obscure transient or localized patterns critical to understanding early disease signatures [25]. Graph theory offers a powerful alternative, enabling the extraction of topological and temporal features from spike relationships without reducing the data to population averages [6]. Graphs can provide a quantitative means of learning both spatial and temporal connections among electrode activities. By modeling the functional connectivity between electrodes [23], it becomes possible to reveal subtle, time-resolved deviations in communication patterns, crucial for detecting early alterations associated with a specific disease such as, for example, a neurodevelopmental disorder.

To fully exploit the potential of artificial intelligence in this context and harness the computational power of CNNs [26], we extend our approach by employing graph neural network (GNN) architectures [27] to tackle graph representations of biological neural networks. This methodology enables one to capture complex and heterogeneous relationships, both inter-region (across electrodes) and intra-recording (across time points). Building on these capabilities, we present a novel platform for monitoring and analyzing MEA extracellular recordings, which generates time-varying graph representations and quantitative graph features associated with MEA signal dynamics. This graph-based representation aims to identify distinctive electrical phenotypes linked to specific biological conditions, with a particular focus on early disease-associated signatures in human forebrain organoids (hFOs). To achieve this, we leverage the graph deviation network (GDN) architecture [28], originally designed for detecting structural or functional anomalies in dynamic graphs. Specifically, GDNs learn a directed graph representation by selecting the time-series forecasting error as the loss function. After the GDN-based forecasting model is trained, the GDN parameters are used to extract the connectivity matrix of the MEA recordings. The connectivity matrix enables the construction of a directed graph for each time window, from which a set of graph descriptors is extracted to quantify the functional interaction between electrodes. By leveraging both the high temporal resolution of MEA recordings and these graph-based features, we can train a classification model capable of automatically detecting instantaneous changes in network connectivity, such as reduced clustering or decreased efficiency. These predictions can support the early identification of disease-related alterations and contribute to a more precise assessment of neurological disorder risk.

To validate our methodology, we considered the case study of hFOs in healthy and drug-exposed conditions, grown in a 24-well plate and monitored by a 16-electrode MEA system for 48 h, collected in 4 different acquisition sessions [29]. According to the pioneer work by Jin et al. [27], the assumption is that in hFOs’ valproic acid (VPA) exposure affects the expression of genes involved in neural development, synaptic transmission, oxytocin signaling, calcium, and potassium signaling pathways, i.e., in processes implicated in autism spectrum disorder (ASD). In fact, genes affected by VPA importantly overlapped with those dysregulated in brain or organoids derived from ASD patients, known as ASD risk genes. Here, we show that MEA analysis further identified that VPA exposure in hFOs disrupts synaptic transmission, causing loss of efficiency, increased node path length, and decreased incoming node connections. We demonstrate that the novel proposed strategies can be coupled with MEA recordings to confirm single-cell RNA sequencing analysis performed in Ref. [29] over biological samples under investigation.

## Materials and Methods

### Experimental design

**Fig. 1.**
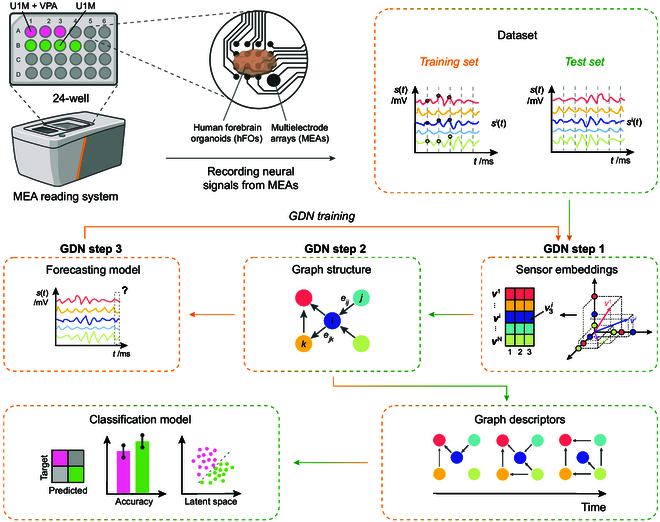
Schematic overview of the platform. A sketch of the whole methodology for the continuous monitoring of the organoid synaptic activity through the analysis of neural activity from hFOs using MEA and a GDN model. Neural signals are recorded from organoids cultured in 24-well plates in treated and untreated conditions (e.g., U1M and U1M + VPA) using MEA reading systems. The recorded voltage signals are split into training and test datasets. The GDN training (orange path) involves 3 steps: (1) sensor embeddings are computed to represent the activity of individual electrodes; (2) a graph structure is learned to model dynamic inter-electrode relationships; and (3) a forecasting model predicts future neural signals. Once the GDN is trained, the temporal graph descriptors of the training set are used to build the classification model then validated using the descriptors obtained by test datasets (green path).

Fig. 1 illustrates the experimental platform developed for monitoring functional connectivity in hFOs. The goal of the study was to assess whether VPA exposure induces early alterations in network-level activity of hFOs, as a model for ASD-linked synaptic dysfunction.

To implement this, we used a 24-well MEA plate (Axion Biosystems) to simultaneously monitor hFOs under different biological conditions. Seven organoids derived from male donors were cultured individually in separate wells. According to the protocol detailed in Ref. [29], VPA was applied at a concentration of 1 mM to 3 wells after 7 days from MEA plating, while the remaining 4 wells served as untreated controls. MEA recordings were performed at 4 time points: 0 h (before treatment), 30 min, 24 h, and 48 h post-VPA exposure, using a sampling rate of 12.5 kHz. For each recording session lasting 5 min, the first 2.5 min was used for training the GDN model, and the remaining 2.5 min was used for testing.

The collected electrophysiological data were processed using a GDN framework. The processing pipeline consists of several key steps, which are briefly summarized here:

1. Each multichannel time series is first encoded into a set of latent embedding vectors.

2. An adjacency matrix is derived from the dot products of these embeddings to represent electrode-to-electrode relationships.

3. A time-varying graph structure is constructed from the adjacency matrix, capturing dynamic connectivity patterns.

4. The forecasting model is trained to optimize the graph effectiveness by minimizing the prediction error between expected and observed signals.

To evaluate the trained GDN, each test dataset was segmented into temporal windows and transformed into graph instances. From each graph, a set of topological descriptors was extracted to quantify the evolving network dynamics. These descriptors were then used as input features for a linear discriminant analysis (LDA) model, which was trained to classify and distinguish the biological conditions across wells based on their connectivity signatures, as shown in Fig. 1.

In the following, we will provide details of the different blocks of the platform.

### Brain organoid culture

Two hiPSC lines respectively derived from one healthy male (U1M, 37 years) and one healthy female (U2F, 28 years) were obtained from CellAPY Technology (Beijing, China). The U1M and U2F hiPSC lines (passage < 30) were used for generating hFOs based on published methods [29]. According to the literature, VPA at 1 mM was considered as a clinically relevant concentration [30] since it was reported to exert nominal effects on neuron differentiation with minimal cell toxicity [31]. Therefore, hFOs were plated in MEA plates and treated with or without 1 mM VPA (Selleck, S1168). According to the preliminary results shown in the pioneer paper of this study, we decided to use only the U1M organoids that display enhanced sensitivity to the drug in the context of synaptic connectivity changes.

### MEA acquisition

MEA Axion Biosystems, a 16-electrode assay, was used to assess whether VPA exposure affects synaptic transmission in hFOs. Since Matrigel-embedded organoids could not sufficiently make contact with the MEA electrodes, non-Matrigel embedded hFOs were generated using the STEMdiff Ventral Forebrain Organoid Differentiation Kit (STEMCELL Technologies, 08630), which was based on the methods described in Ref. [32]. hFOs aged 42 days were seeded into the MEA culture wells. After 7 days of culture, hFOs were treated with or without 1 mM VPA as indicated before. Considered electro activities were recorded at 0 h, 30 min, 24 h, and 48 h post-VPA exposure. Each well contained a different biological condition or replica. In particular, for this work, wells A1 to A5 include U1M + VPA (pink wells in Fig. 1), wells B1 to B5 include U1M (green wells in Fig. 1). Each produced a multivariate time series composed of 16 signals over 5 min. For reproducibility aspects, we finally considered A1 to A3 (U1M + VPA) and B1 to B4 (U1M) wells for the analysis.

The 16-MEA raw signals have been acquired at 12.5 kHz for a period of 5 min (about 3.75e6 samples, with a time resolution of 0.08 ms).

### MEA recordings preprocessing

**Fig. 2.**
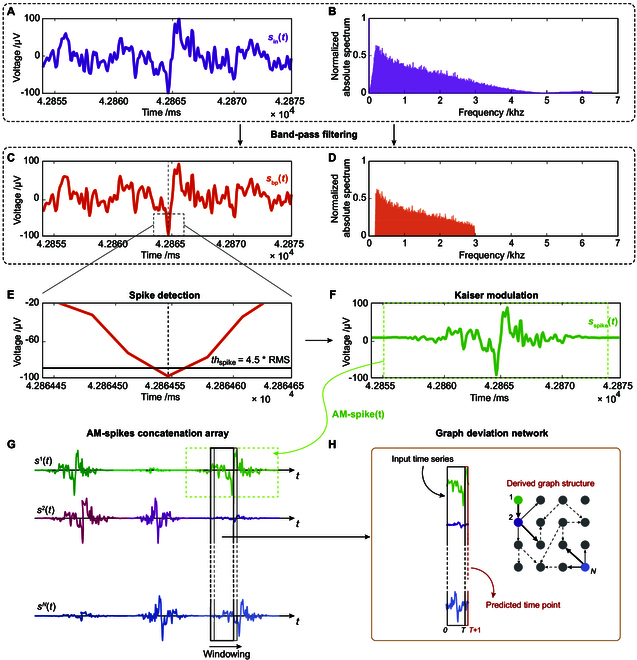
A summary of the main steps in the pre-processing phase. (A) A segment of the original signal from electrode 13. (B) Its Fourier spectrum. (C) The band-pass Butterworth filtered signal [200 to 3,000] Hz. (D) Fourier spectrum of the filtered signal. (E) Detected spike through threshold *th*_spike_ = 4.5*RMS, where RMS is computed as the average values of window-based standard deviation computation with windows of duration 50 ms. (F) The Kaiser-windowed modulated spike segment, width = 20 ms, and selection of centered 18 ms. (G) Amplitude modulated (AM)-Spike signals concatenation along the time axis for each channel (electrode) and segmentation in sub-sequences of 4.5 ms (*T* = 56 time points). (H) GDN block, subsequences set of 56 time points as input, one following predicted time point, and corresponding derived graph structure.

Each signal *s*_in_(*t*) is pre-processed as follows:

1. Apply band-pass filtering in the range [200 to 3,000] Hz using a zero-phase band-pass Butterworth filter with a stopband attenuation of 60 dB. Indicate with *s*_bp_(*t*) the corresponding band-pass multivariate time series. An example of the signal extracted before and after filtering is shown in Fig. 2A and C. Fig. 2B and D show the magnitude of the Fourier spectrum of signals in Fig. 2A and C, respectively.

2. Apply a spike detection procedure based on the algorithm described in Ref. [7]. The spike detection algorithm first estimates the baseline noise level by identifying “spike-free” periods in the signal. The signal is divided into 50-ms windows. The procedure is as follows:

• The standard deviation (SD) is calculated for each 50 ms time window.
• The median (MD_SD_) of all SD values is then computed, as it is more robust than the mean against possible outliers. This value represents the noise level.
• Then, the average of the SD values exceeding the MD_SD_ is calculated; this is referred to as the ref-value*,* and it is attributed to signal rather than noise, since it is high enough with respect to the noise level.
• Spikes are defined as absolute signal amplitudes greater than the threshold *th*_spike_ = 4.5 ref-value. The factor 4.5 is chosen heuristically but is progressively decreased until at least one spike is detected in the entire electrode signal. The spike location procedure is shown in the zoomed-in image in Fig. 2E.

3. Given the threshold value *th*_spike_, time points corresponding to the spike are detected and a post-processing is then applied to *s*_bp_(*t*) to obtain the spike-modulated time series, *s*_spike_(*t*). Specifically, whenever a spike is detected in the signal from one electrode, we multiply the band-pass filtered signal of that electrode, as well as the signals from all other electrodes at the same time point, by a Kaiser window with a shape factor of 10, centered on the detected spike and spanning a time window of approximately 20 ms (see an example of the modulated signal in Fig. 2F).

This procedure emphasizes network activity around the detected spike and highlights correlations across different electrodes, although it may partially obscure spikes occurring at slightly different times in other electrodes. Because the analysis focuses on the modulation of collective activity around reference events, this approach does not hinder the capture of relevant network dynamics. Based on previous investigations [9], we assumed that the raw signal surrounding the spike provides additional relevant information about the electrode activity. Therefore, after amplitude-modulating the spike, we selected 18-ms intervals (AM-spikes, as in the green dashed box in Fig. 2F), removing low-significance tails and retaining waveforms that extended beyond the spike event itself.

4. All the AM-spike signals from each electrode (225 time points each) are then concatenated sequentially in chronological order, so that for every detected spike, the corresponding AM-spike signals are placed one after the other along the time axis. This operation is repeated for all channels, thereby creating a sort of AM-spike raster plot (as in the classical raster plot, a simultaneous extraction from all the electrodes is implemented). An example of the results after concatenation across all channels is shown in Fig. 2G. The Kaiser modulation is beneficial not only for shaping the spike waveform but also for ensuring a smooth transition at the edges of the concatenated segments. By centering the window at the detected spike time point and tapering the signal to zero at the window’s boundaries, the modulation prevents artifacts that might be misinterpreted as spurious spikes. In this way, a new multivariate sequence is constructed and used as input to the GDN. Specifically, the multivariate time series is segmented into overlapping sub-sequences of duration 4.5 ms (corresponding to *T* = 56 time points), with a sliding window that advances by one time point at each step as indicated by the black dashed rectangular boxes in Fig. 2G. Then, each sequence is sent as input to the GDN architecture to extract the graph corresponding to the subsequent time point (Fig. 2H). According to the number of spikes detected, the total number of sequences sent to the GDN for training can vary. Additionally, it is important to note that, from each AM-spike signal consisting of 225 points, we can extract a total of 169 graph structures, due to the 56-time-point latency required for the construction of a single graph at time *t*. Indeed, in the signal prediction task, reconstructing the signal at time point *t* requires the preceding *T* = 56 samples. Consequently, the first graph can only be built at time point 57, since all preceding samples are needed to initialize the prediction window. From that point onward, graphs are generated with a sliding step of 1 sample. As a result, the first 56 time points cannot be directly mapped into graphs and are effectively excluded from the graph sequence.

For this reason, the duration of each sub-sequence (4.5 ms, corresponding to *T* = 56 time points) was chosen as a compromise between temporal resolution and dynamical context. The window is short enough to avoid losing too many time points during the prediction step, thereby preserving the integrity of the spike peak, yet long enough to include sufficient surrounding dynamics to provide the model with the temporal context required to reconstruct the signal accurately.

Numerical details will be provided in the following.

To ensure a validation set during the learning phase, we segment the first half of the time series (around 2.5 min) for the training dataset and do the same for validation on the remaining half of the time series. A different GDN is trained in each well of interest, leading to the construction of 24 different trained GDN architectures, later indicated with $\begin{array}{c} \mathrm{GDN}\left(n,\;m\right),\;n=1,\ldots ,4\;\text{and}\;m=1,\ldots ,6 \end{array}$. . Table 1 lists the training parameters used in the learning phase of all the wells. By limiting the analysis to wells (A1 to A3 and B1 to B4), only the corresponding GDN models will be used in this work.

**Table 1.** Training parameters used during the GDN learning phase

Name of the parameters	Value
Number of epochs	10
Learning rate	1e−3
Embedding dimensions	128
Number of hidden units in fully connected layer	12
Initialization *μ* for embedding vectors	0
Initialization *σ* for embedding vectors	1e−3
Minibatch size	200
Optimization method [57]	ADAM

### Graph deviation network

**Fig. 3.**
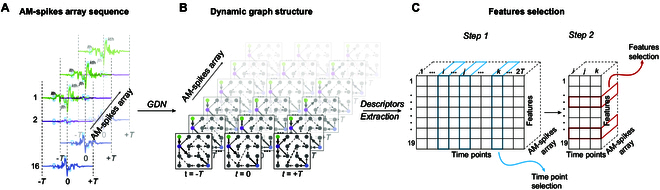
A summary of feature extraction and selection. (A) Temporal series of AM-spikes arrays, simultaneously recorded from 16 electrodes. (B) Corresponding dynamic graph structures extracted through the GDN, followed by descriptor extraction. (C) Feature selection process: Step 1, selection of the most discriminative time points (above the 95th percentile, in blue) considering them as shared temporal index; Step 2, from the selected time points, individual graph descriptors are evaluated through the selection of the most important features (with DS score greater than 0.75, in red) to build the classification model input.

GNN architecture has emerged as a powerful tool for learning non-Euclidean data representations [27], paving the way for modelling real-world time-series data. This new approach enables the estimation of both inter-variable (connections between different electrodes within a multivariate series) and intra-temporal (dependencies between different time points in a given series) interplay. Among the plethora of existing architectures and possible applications, in this work, we refer to a specific version of the GNN, named GDN [28]. As shown in Fig. 1, GDN first encodes a low-level representation of each time series for a given observation window using embedding vectors. Next, a connectivity matrix is computed as the dot product of the embedding vectors, resulting in the extraction of a graph representation. Finally, forecasting architecture is trained by minimizing forecasting error loss function. After the training process, GDN can encode each multivariate time series of fixed duration into a directed graph structure (Fig. 3A and B).

Consider a training dataset consisting of output signals from *N* electrodes over *T* time ticks *t*: the AM-spikes array at time *t* is denoted as $\;s\left(t\right)\in R^{N}=\left[s^{1}\left(t\right),\ldots ,\;s^{N}\left(t\right)\right]$ , where $s^{i}\left(t\right)$ is the *i*th electrode output. According to the architecture shown in Fig. 1, the GDN construction is based on the following main blocks: (a) sensor embedding definition; (b) graph structure learning; (c) graph attention-based forecasting, and (d) graph deviation scoring. Each step is examined in detail below.

#### Sensor embedding

A GDN has the potential to learn a low-dimensional representation of each time series $s^{i}\left(t\right)$, namely, $v^{i}\in$ $\;R^{d}=\left[v_{1}^{i},\ldots ,v_{d}^{i}\right]\;$, with *d* << *T*, called embedding vectors. Such a representation allows one not only to measure similarity between pairs of electrodes in each time interval, even in the presence of heterogeneous signals, but also to easily identify neighbors in the graph structure, i.e., electrodes that maximally mutually interact. The embedding vector $v^{i}$ [corresponding to the electrode signal $s^{i}\left(t\right)]$ is randomly initialized and learned during the training phase. Considering Fig. 1, the embedding array is a vector in a *d*-dimension space.

#### Graph structure learning

The major goal of the learning phase is to derive a directed graph representation of the relationships among electrodes. A directed graph is used where each node represents an electrode and each edge from node *i* to node *j* represents the relationship between electrode *i* and electrode *j* (see Fig. 1). An edge directed from node *i* to node *j* indicates that the electrode output *i* is used to model the behavior of the electrode output *j*. In this way, by not forcing symmetry in the relationships between pairs of electrodes, we are able to model causality. A corresponding asymmetric adjacency matrix $A_{ij}$ is hence derived.

Dependencies between the *i*th node and its candidates j∈[1,...,i-1,...,i+1,....N] is computed by the cosine similarity (i.e., the dot product) between corresponding embedding vectors $v^{i}$ and $v^{j}$ by

$$e_{\mathrm{ji}}=\frac{{\left(v^{i}\right)}^{⊥}v^{j}}{\left\|v^{i}\right\|\cdot \left\|v^{j}\right\|}$$ (1)

Clarifying cosine similarity, as a stand-alone static similarity metric, is not inherently related to causality. In our framework, however, it is used as part of a learned mechanism within a GDN trained for time-series prediction. During training, the model learns to attend to nodes whose past states improve the prediction of others, meaning that cosine similarity encodes predictive dependencies. Thus, while cosine similarity is not inherently causal, it becomes a meaningful proxy for temporal dependency through the model’s learning objective function.

With respect to the original formulation in Ref. [24], we decided to maintain all the electrodes, 16 in the considered MEA platform, letting the learning phase assign different weights to each electrode without the a priori limitation of the maximum number of electrodes to consider. The intrinsic heterogeneity of the organoids under monitoring in the multi-well platform does not allow us to foresee how many electrodes can be simultaneously involved in organoid electrical activity monitoring, also by virtue of the fact that in low-density planar MEA systems, electrodes collect extracellular recordings that represent a cumulative response of a multitude of neurons.

#### Graph attention-based forecasting

The GDN is thought to detect anomalous behavior in each electrode. To accomplish this task, a forecasting-based approach is used as an objective function for the learning phase [28]. To do this, first, we define a sequence of past *w* values for each electrode signal $s^{i}\left(t\right)$, defining the new time series $x^{i}\left(t\right)$:

$$x^{i}\left(t\right)=\left[s^{i}\left(t-w\right)\;s^{i}\left(t-w+1\right)\;\ldots \;s^{i}\left(t-1\right)\right]$$ (2)

that are used to predict the value $s^{i}\left(t\right)$.

To combine the past values in order to create a predictive model of the future value $s^{i}\left(t\right)$, a learnable feature extraction procedure is applied.

First, the $x^{i}\left(t\right)$ vector values are aggregated as follows:

$$z^{i}\left(t\right)=\text{ReLu}\left(\alpha_{i,i}Wx^{i}\left(t\right)+\sum_{j}\alpha_{i,j}Wx^{j}\left(t\right)\right)$$ (3)

where $W\in R^{d+w}$ is a trainable weight matrix that applies a shared linear transformation to every node, $\alpha_{i,i}$ are attention coefficients that quantify the contribution of neighbor *j* to node *i*, and ReLu is the activation function.

#### Attention mechanism

Our model uses a graph attention mechanism to weigh the importance of information from neighboring nodes when computing each node’s feature representation. The attention coefficients $\alpha_{i,j}$ are computed through the following 3 steps:

First step

$$g^{i}\left(t\right)=v^{i}⊕Wx^{i}\left(t\right)$$ (4)

where $v^{i}\in R^{d}$ is the static embedding vector that encodes the structural role of node *i*. It does not change over time and provides a prior representation of the node; $Wx^{i}\left(t\right)$ is the dynamic feature vector, obtained by linearly transforming the recent time-windowed signal of node *i*. It captures the time-varying state of the electrode at time *t.*

From Eq. (3), ⊕ denotes concatenation of vectors, leading to a vector $g^{i}\left(t\right)\in$ $R^{2d}$, in which the first half of the vector provides static (node-specific) information and the second half encodes the dynamic (signal-dependent) state.

Second step

The raw attention score between nodes *i* and *j* is then calculated as:

$$\begin{array}{c} \pi \left(i,j\right)=\text{LeakyReLU}\left(a^{⊥}\left(g^{i}\left(t\right)\;⊕\;g^{j}\left(t\right)\right)\right) \end{array}$$ (5)

where a∈ $R^{4d}$ is a vector of learnable attention weights, $\left(g^{i}\left(t\right)\;⊕\;g^{j}\left(t\right)\right)\in R^{4d}$ is the concatenation of transformed embeddings of nodes *i* and *j*, and LeakyReLU [33] is a type of activation function based on a ReLU, but with nonnull slope for negative values.

Third step

Finally, the attention coefficients are obtained through soft-max normalization [28,33]:

$$\alpha_{i,j}=\frac{\text{exp}\left(\pi \left(i,\;j\right)\right)}{\sum_{k=1}^{N}\text{exp}\left(\pi \left(i,\;k\right)\right)}$$ (6)

which ensures $\alpha_{i,j}>0,$ and $\sum \alpha_{i,j}=1.$ The attention coefficients form the adjacency matrix to derive the graph structure.

By element-wise multiplying (∘) the features vector $z^{i}\left(t\right)$ from Eq. (3) with the embedding vectors $v^{i}$, and then introducing a fully connected layer with coefficients $f_{\theta }$, we obtain the estimation of the *N*-dimensional output vector as follows:

$$\hat{s}\left(t\right)=f_{\theta }\left(v^{i}\;\cdot \;z^{i}\left(t\right)\right)$$ (7)

By using the mean squared error objective function, defined as

$$L_{\mathrm{MSE}}=\frac{1}{T-w}\sum_{t=w+1}^{T}{\left\|\hat{s}\left(t\right)-s\left(t\right)\right\|}^{2}$$ (8)

GDN finally assigns the learnable parameters $v^{i}$, W, $a^{⊥}$, and $f_{\theta }$ [28].

The trained GDN is then used in the following graph-based feature monitoring procedure.

### Graph-based descriptors

The study of complex brain networks using graph theory goes back to the introduction of the “Human Connectome” and, even before, by the identification of so-called “small-world” architecture [34], the result of a natural process to satisfy the balance between minimizing the resource cost and maximizing the flow of information among the network components [35–40].

Theoretical examinations, supported by experimental investigations [41–43], have pointed out that the brain regions are more likely to interact with their neighboring areas to reduce the whole metabolic costs, while, at the same time, they need to have a small number of long-distance connections among themselves to accelerate data transmission [44,45]. Centrality measures are among the most relevant tools to provide a quantitative representation of such behaviors. Centrality is a crucial concept in graph theory that deals with distinguishing nodes that are important or central among the whole list of other nodes in a graph. By considering each node individually and coupling this aspect with the role of the other neighboring nodes in the graph, different kinds of centrality measures can be defined and will be used in this work. In light of these considerations, with the aim to monitor the connectivity and the causality between pairs of electrodes (regions of the brain organoid) over time, we extracted standard centrality measure descriptors according to state-of-the-art algorithms [46] from the learned GDN graph representation.

1. Degree centrality: Indegree $\text{InDEG}\left[P_{i}\right]$ and Outdegree $\text{OutDEG}\left[P_{i}\right]$

These scores simply calculate for each node $P_{i}$ the number of input and output links. For each descriptor we extracted: $\;\underset{i}{\text{mean}}\;\text{InDEG}\left[P_{i}\right]$, $\underset{i}{\mathrm{SD}}\;\text{InDEG}\left[P_{i}\right]$, $\min_{i}\;\text{InDEG}\left[P_{i}\right],\max_{i}\;\text{InDEG}\left[P_{i}\right]$, $\underset{i}{\text{mean}}\;\text{OutDEG}\left[P_{i}\right]$, $\underset{i}{\mathrm{SD}}\;\text{OutDEG}\left[P_{i}\right]$, $\min_{i}\;\text{OutDEG}\left[P_{i}\right],\;\underset{i}{\max}$ $\text{OutDEG}\left[P_{i}\right]\;$. The higher the degree, the more crucial the node is in the graph. It is one of the simplest metrics among the centrality measures of node connectivity.

2. Closeness centralities: $\text{InCLOSE}\left[P_{i}\right]$ and $\text{OutCLOSE}\left[P_{i}\right]$

Closeness centrality identifies a node’s importance based on how close it is to all the other nodes in the graph. The closeness is also known as geodesic distance (GD), which is the number of links included in the shortest path between 2 nodes. Numerically,

$$\begin{array}{c} \text{InCLOSE}\left[P_{i}\right]=\frac{1}{\sum_{j\neq i,P_{j}\to P_{i}\;}\mathrm{GD}\left(P_{i},P_{j}\right)} \end{array}$$ (9)

$$\begin{array}{c} \text{OutCLOSE}\left[P_{i}\right]=\frac{1}{\sum_{j\neq i,P_{i}\to P_{j}}\mathrm{GD}\left(P_{i},P_{j}\right)} \end{array}$$ (10)

where $P_{j}\to P_{i}$ means all node *j*s that link node *i*, while $P_{i}\to P_{j}$ indicates all nodes *j*s reachable from node *i*. As above, for each of the parameters extracted, we calculate $\underset{i}{\text{mean}}\;\text{InCLOSE}\left[P_{i}\right],$ $\begin{array}{c} \begin{array}{c} \underset{i}{\mathrm{SD}}\;\text{InCLOSE}\left[P_{i}\right],\;\min_{i}\;\text{InCLOSE}\left[P_{i}\right], \end{array} \end{array}$ $\begin{array}{c} \underset{i}{\text{max }}\;\text{OutCLOSE}\left[P_{i}\right] \end{array}$, $\begin{array}{c} \begin{array}{c} \underset{i}{\text{mean}}\;\text{OutCLOSE}\left[P_{i}\right],\;\underset{i}{\mathrm{SD}}\;\text{OutCLOSE}\left[P_{i}\right], \end{array} \end{array}$ $\;\min_{i}\;\text{OutCLOSE}\left[P_{i}\right]$, and $\max_{i}\text{OutCLOSE}\left[P_{i}\right]$ .

3. Betweenness centrality*:* $\text{Betw}\left[P_{i}\right]$

Betweenness centrality defines the importance of any node based on the number of times it occurs in the shortest paths, *sp*, between other nodes. It measures the percentage of the shortest path in a network and where a particular node lies in it. A node with high betweenness centrality is considered the most influential one over other nodes in the network. In the formula, $\text{Betw}\left[P_{i}\right]$ is given by

$$\text{Betw}\left[P_{i}\right]=\sum_{j\neq i\neq k\in V}\frac{{\mathrm{sp}}_{\mathrm{jk}}\left(P_{i}\right)}{{\mathrm{sp}}_{\mathrm{jk}}}$$ (11)

where *V* is the set of nodes in the graph, ${\mathrm{sp}}_{\mathrm{jk}}$ is the total number of shortest paths between nodes *j* and *k* not including node *i*, and ${\mathrm{sp}}_{\mathrm{jk}}\left(P_{i}\right)$ represents the total number of shortest paths between nodes *j* and *k* including node *i*. As above, we extracted the following statistical descriptors: $\underset{i}{\text{mean}}\;\text{Betw}\left[P_{i}\right]$, $\underset{i}{\mathrm{SD}}\;\text{Betw}\left[P_{i}\right]$, $\min_{i}\;\text{Betw}\left[P_{i}\right]$, and $\max_{i}\;\text{Betw}\left[P_{i}\right]$.

4. Page rank: $\mathrm{PR}\left[P_{i}\right]$ [47])

$$\mathrm{PR}\left[P_{i}\right]=\frac{1-d}{N}+d\left(\sum_{k=1,k\neq i}^{n}\frac{\mathrm{PR}\left[P_{k}\right]}{C\left[P_{k}\right]}\right)$$ (12)

where $P_{i}$ is the node of which we calculate the page rank, *N* is the total number of nodes, *d* is an empirical constant equal to 0.85, $P_{k}$ is the total number of nodes linking *A*, PR[$P_{k}$] is the page rank of each node $P_{k}$, and *C*[$P_{k}]$ is the total number of links starting from node $P_{k}.$

In a few words, the page rank provides the sum of the frequency of links from each node rather than the *i*th, that links the node *i* with respect to the total number of links existing from the node *k*. A node with a high rank is a node for which most of the links from nodes rather than the *i*th links to the *i*th node, a sort of exclusive relationship. Given that we have a page rank index for each electrode $\mathrm{PR}\left[P_{i}\right]$, we extract the following statistical values $\underset{i}{\text{mean}}\;\mathrm{PR}\left[P_{i}\right]$, $\underset{i}{\mathrm{SD}}\;\mathrm{PR}\left[P_{i}\right]$, $\max_{i}\;\mathrm{PR}\left[P_{i}\right]\;,\text{and}\;\min_{i}\;\mathrm{PR}\left[P_{i}\right]$

5 and 6. Hub rank: $\begin{array}{c} \mathrm{HUB}\left[P_{i}\right]\;\text{and authority}\;\mathrm{rank}:\text{AUTH}\left[P_{i}\right] \end{array}$

The hub and the authority scores derived by the hyperlink-induced topic search algorithm (also known as hubs and authorities) is a link analysis algorithm that rates web pages. The central idea is that a hub is a page that acts as a repository of links, a sort of shunting node, more than an authority node, i.e., a node with many links entering it. A hub node may present a very intense activity without being an end-point node. The dependence between authority and hub ranks motivates the iterative algorithm to fix them. For both the scores, we derived the following statistical values: $\underset{i}{\text{mean}}\;\mathrm{HUB}\left[P_{i}\right]$, $\underset{i}{\mathrm{SD}}\;\mathrm{HUB}\left[P_{i}\right]$, $\;\min_{i}\;\mathrm{HUB}\left[P_{i}\right]\;$, $\begin{array}{c} \;\max_{i}\;\mathrm{HUB}\left[P_{i}\right],\;\underset{i}{\text{mean}}\;\mathrm{HUB}\left[P_{i}\right],\;\underset{i}{\mathrm{SD}}\;\text{AUTH}\left[P_{i}\right] \end{array}$, $\min_{i}\;\text{AUTH}\left[P_{i}\right],\text{and}\;\max_{i}\;\text{AUTH}\left[P_{i}\right]\;$.

7. Global descriptors*.* In addition to the local descriptors (one for each node $P_{i})$, we also extracted global descriptors for the graph learned. Specifically, we computed the following:

Characteristic or average path length $\;\left(\mathrm{CPL}\right)$

$$\mathrm{CPL}=\frac{1}{N\cdot \left(N-1\right)}\sum_{i\neq j\in V}\mathrm{SP}\left(P_{i},P_{j}\right)$$ (13)

which corresponds to the average minimum path between each pair of nodes in the graph, where *N* is the total number of nodes. It is a measure of the efficiency of information transport in a network.

Diameter

Diameter corresponds to the shortest path between the most distant nodes in the graph; diameter measures the extent of a graph.

$$\text{Diameter}=\underset{i\neq j\in V}{\text{max}}\mathrm{SP}\left(P_{i},P_{j}\right)$$ (14)

Note that in the calculation of CPL and of Diameter, infinite paths (so defined a path between 2 nodes not connected through a path) are excluded.

Global Efficiency (Glob-Eff)

$$\begin{array}{c} \text{Glob}-\mathrm{Eff}=\frac{1}{N\cdot \left(N-1\right)}\sum_{i\neq j\in V}\frac{1}{\mathrm{SP}\left(P_{i},\;P_{j}\right)} \end{array}$$ (15)

The global efficiency, also named average communication efficiency of the graph, represents how efficiently the information flows through the network. The underlying idea is that the more distant 2 nodes are in the network, the less efficient their communication will be.

Due to the intrinsic properties of the GDN, some of the 35 parameters exhibited nearly constant values across the dataset and were therefore excluded from the analysis. For instance, in the PageRank definition, the sum of all node values is fixed at 1, which means the average remains constant over time and provides no additional information. Similarly, hub rank and authority rank are normalized by their respective sums across nodes; since the number of nodes remains constant in our application, their averages also do not vary over time. Based on these considerations, we retained only the 19 features that showed temporal variability within each well. The final set of descriptors includes: Diameter, average path-length, min incloseness, max betweenness, std betweenness, mean betweeness, std outdegree, max indegree, min indegree, std indegree, max authrank, min authrank, std authrank, max hubrank, min hubrank, std hubrank, max pagerank, min ragerank, and std pagerank.

Those descriptors effectively represent a condensed form of information originally derived from 16 signals (electrodes). They capture not only the presence of detected spikes, considering also the information surrounding spikes themselves, but also the interconnections among the electrodes, which serve as the nodes of the graph. As a result, the output is a matrix of 19 features across 169 time points for each individual AM-spike array. Feature processing and subsequent classification tasks are performed on single spike array signals, enabling analysis with millisecond resolution.

### Feature processing and selection

We calculated the descriptors for each of the 4 acquisition sessions, 0 h, 30 min, 24 h, and 48 h. To account for residual noise in the descriptors computations, and the inter-replicas heterogeneity of hFOs, we performed the following normalization. By indicating each sequence of descriptors collected as $X_{U1M}^{0\;h}\left(t\right)$, $X_{U1M}^{30\;\min}\left(t\right),\;X_{U1M}^{24\;h}\left(t\right)$, $X_{U1M}^{48\;h}\left(t\right),\;X_{U1M+\mathrm{VPA}}^{0\;h}\;\left(t\right)$, $\;X_{U1M+\mathrm{VPA}}^{30\;\min}\;\left(t\right),\;X_{U1M+\mathrm{VPA}}^{24\;h}(t)$ , and $X_{U1M+\mathrm{VPA}}^{48\;h}\left(t\right)$ , we performed a baseline normalization, by dividing each sequence of descriptors at time 30 min, 24 h, and 48 h by the mean value measured at time 0 h (i.e., that we indicated as baseline) in the same well, obtaining normalized data sequences indicated as ${\hat{X}}_{U1M}^{30\;\min}(t)$, ${\hat{X}}_{U1M}^{24\;h}(t)$, ${\hat{X}}_{U1M}^{48\;h}\left(t\right)$, ${\hat{X}}_{U1M+\mathrm{VPA}}^{30\;\min}(t)$, ${\hat{X}}_{U1M+\mathrm{VPA}}^{24\;h}(t)$, and ${\hat{X}}_{U1M+\mathrm{VPA}}^{48\;h}\left(t\right)$. Therefore, we did not consider for the analysis the values collected at time 0 h. In this way, we are confident about the reduction of the heterogeneity of the hFOs’ growth process that is observed even in nominally identical conditions.

The normalized descriptors are then used to construct a unique classification model (over all the wells) for the recognition of the effects of VPA exposure over hFO synaptic activity. With the aim of deriving a robust and effective classification model, the descriptors have been preliminarily analyzed in terms of their capability to recognize discriminative patterns between VPA exposure and control samples. We implemented area under the ROC (receiver operating characteristic) curve (AUC) method to plot the classification capabilities of each descriptor. Features with sufficiently high AUC values (or sufficiently low) are then used in the construction of the prediction model. In particular, as defined in a previous work [48], we define a discriminant score (DS) for each feature *X* as follows:

$$\mathrm{DS}\left(X\right)=\text{max}\left(\mathrm{AUC}\left(X\right),1-\mathrm{AUC}\left(X\right)\right)$$ (16)

The value of DS is in range [0.5 to 1], where 0.5 is the random guess indicating poor discrimination capability and 1 represents perfect discrimination capability. As discussed in Ref. [46], DS values are preferred over the AUC values because they enable the use of a unilateral decision rule.

To assess the discriminative power of each feature in the VPA classification task, we calculated the DS for every feature at each time point. Time points (from *t* = 0 to *t* = 169) were treated as a shared temporal index across all the time series. That is, we first evaluated all features at *t* = 0 across all series, then repeated the process for *t* = 1, and so on. This approach allowed us to identify time points that were globally most discriminative. We then selected the time points whose DS values exceeded the 95th percentile of the overall DS distribution (see Step 1 in Fig. 3C). From these high-scoring selected time points, we further retained only the most informative features, specifically, those with a DS greater than 0.75, defining them as the most informative features (Step 2 in Fig. 3C).

The ability to monitor graph descriptors for each organoid at the millisecond level enables us not only to track the dynamics of synaptic connectivity across different neuronal culture regions but also to identify specific temporal intervals around spike events where the effects of VPA exposure are more pronounced. Finally, the selected features represent, for each spike event, the input data used in the VPA classification model for the prediction.

### Classification model design

The application context considered reveals 2 levels of heterogeneity. First, each organoid displays substantial inter-well variability, which we mitigated by normalizing the descriptors with respect to the mean values obtained in session 0 h. Second, heterogeneity also arises across sessions of the same organoid, primarily as a consequence of aging and the resulting decline of synaptic activity. To optimize the classification model design and test, we performed a cross-validation procedure by proceeding as follows:

1. We trained a distinct GDN model for each organoid at each session and extracted the corresponding graph features. Each feature matrix is then composed of as many rows as the number of time frames we have in the AM-spike concatenated signal and 19 columns. We have a feature matrix for each well (1 to 7) and each session (0, 30 min, 24 h, and 48 h).
2. We then vertically concatenated all the feature matrices for each session, leading to a matrix with as many rows as the time frames of all the AM-spike concatenated signals for each organoid by the number of wells. We obtained a different feature matrix for each session. Session at 0 h was considered only for the sake of normalization and therefore will not be considered in the analysis. Numerical details of the dimensions of each feature matrix are provided in the “Data analysis” section.

Thus, we designed an LDA model for the classification task [49]. To cross-validate the classification model based on graph descriptors, we implemented a leave-one-well-out (LOWO) cross-validation strategy, training the model on 6 wells and testing in turn the remaining well, for each session. The procedure was repeated for each well. In this way, we are confident to test every segment of every MEA recording in an unbiased way. Classification results were assessed through the accuracy of classification, computed separately for each session of acquisition. In this way, we were able to evaluate whether there was a temporal VPA effect on organoid synaptic response.

By classifying each temporal segment independently, this approach can assign different labels across time, which is particularly useful under nonstationary conditions when, for example, capturing instantaneous abnormal events, monitoring responses during dynamic drug administration, or tracking changes throughout organoid growth. We also tested the predictive capability of the classification model trained over early sessions (30 min) over the 24-h and the 48-h sessions.

## Results

To validate the proposed monitoring platform, we analyzed data from MEA experiments involving hFOs. This experiment investigated the association between prenatal exposure to VPA and increased risk of ASD [29]. In addition to functional assessments, a study [29] identified VPA-induced alterations in gene expression and signaling pathways relevant to neurodevelopment.

Further details of the protocol used can be found in Ref. [29]. By using the proposed platform, we aim to demonstrate the existence of temporally confined events that are derived from the spike activity and that are relevant for the precise characterization of the efficiency in the communication between different compartments of the organoids. In particular, we assumed that the raw signal surrounding a spike contains additional relevant information about electrode activity and that MEA data can be analyzed not only by examining individual spikes but also by considering them collectively, i.e., AM-spikes and AM-spikes array. This approach enables the decomposition of the signal into multiple informative components. In the following, we show the main results achieved by using the mentioned approach.

### Data analysis

The analysis was conducted on the 7 different wells each containing an organoid and an experimental condition. Four of them included an organoid with no treatment (B1 to B4 in Fig. 1), which we named CTRL. Three of them contained organoids exposed to VPA (A1 to A3 in Fig. 1), which we named VPA. Each well has been individually characterized in terms of AM-spike segments, each encoded as graph descriptors, leading to a very large amount of data. Table 2 reports the number of segments collected and encoded for each well. The high number demonstrates the validity of the results presented. In addition, the heterogeneity of the numbers demonstrates the inter-organoid variability and reinforces the necessity to normalize descriptors for the baseline values acquired at time 0 h, to avoid any bias.

**Table 2.** Number of AM-spike segments analyzed in each session of acquisition for each well

No. of AM-spike segments analyzed	0 h	30 min	24 h	48 h
Well (B1)—CTRL	143,481	136,383	114,244	136,383
Well (B2)—CTRL	143,988	137,228	101,062	139,763
Well (B3)—CTRL	148,889	104,949	141,453	125,398
Well (B4)—CTRL	97,682	54,080	99,879	62,868
Well (A1)—VPA	143,312	115,258	135,876	135,876
Well (A2)—VPA	71,656	68,783	125,567	123,032
Well (A3)—VPA	137,904	64,051	142,805	98,358

### GDN forecasting performance

To assess the GDN performance in the forecasting task, which indirectly reflects its ability to characterize the graph architecture underlying the synergistic activity of the electrodes, we evaluated the prediction results obtained when the trained GDN was applied to the test sequences. For this purpose, each recording was divided into 2 parts: The first half of the time series was used for GDN training, and the second half was used for testing. Table 3 reports the values of the normalized root mean square error (NRMSE, i.e., RMSE normalized by the standard deviation of the expected signal, to reduce sensitivity to spike amplitudes) and the maximum cross-correlation (sensitive to spike synchronization). For both metrics, we computed the median and the median absolute deviation (MAD) across the wells of each condition. Results are reported for the training sequence and for the test sequences separately. As expected, no temporal shift was observed between the predicted and the actual signals.

**Table 3.** GDN forecasting errors for the training and test sets across the 4 sessions. Results are separately averaged (median values) over CTRL and VPA wells. Median absolute deviation (MAD) values are included in the parentheses.

Normalized root mean square error		Median (median absolute deviation) values			
		0 h	30 min	24 h	48 h
Well (B1–B4)—CTRL	Training	0.394 (0.026)	0.476 (0.028)	0.440 (0.046)	0.449 (0.026)
	Test	0.412 (0.012)	0.460 (0.023)	0.462 (0.043)	0.456 (0.038)
Well (A1–A3)—VPA	Training	0.397 (0.052)	0.447 (0.101)	0.329 (0.101)	0.366 (0.084)
	Test	0.417 (0.028)	0.451 (0.058)	0.329 (0.106)	0.336 (0.060)
Maximum cross-correlation		Median (median absolute deviation) values			
		0 h	30 min	24 h	48 h
Well (B1–B4)—CTRL	Training	0.845 (0.032)	0.796 (0.047)	0.834 (0.055)	0.853 (0.024)
	Test	0.820 (0.027)	0.758 (0.068)	0.822 (0.028)	0.811 (0.042)
Well (A1–A3)—VPA	Training	0.809 (0.036)	0.757 (0.107)	0.898 (0.068)	0.897 (0.039)
	Test	0.764 (0.024)	0.697 (0.052)	0.898 (0.178)	0.890 (0.044)

### Feature selection results

**Fig. 4.**
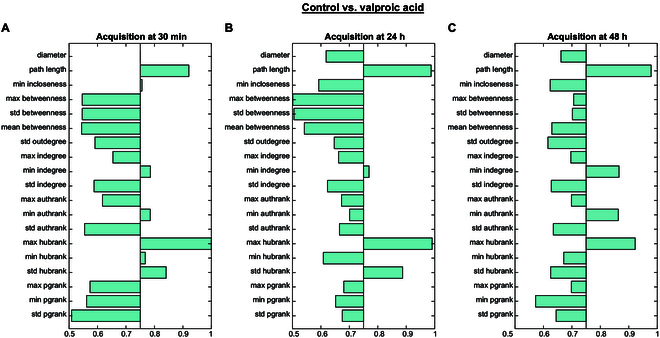
DS values computed for features extracted. Features discrimination capability for sessions (A) 30 min, (B) 24 h, and (C) 48 h using a visualization threshold of 0.75.

To gain deeper insight into the information conveyed by individual graph features at specific time points, we first analyzed the DS across the 3 acquisition sessions. Fig. 4 reports the DS values calculated for all 19 features considering all the time points, using a visualization threshold of 0.75. Notably, some features, such as path length, consistently exhibit high DS values across all sessions, indicating an overall relevance. Conversely, other descriptors show an important contribution only in specific sessions (i.e., diameter at 30 min).

As shown, values are quite low and just a bit larger for the test set, as expected. MAD values are also very low, indicating a small variation of the performance over the wells of the same condition. Ultimately, we observe a general decrease in the prediction performance for VPA condition with respect to CTRL, which can be justified by a decreased level of predictability due to the acid effect on the synaptic activity. No relevant effect is observed as session goes on.

To build a robust classification method, we have considered a 2-step feature selection. Firstly, we used the DS metric to identify the most discriminative time points by evaluating their overall significance across all time series of descriptors over the 3 sessions. This strategy allowed us to focus on time points at which feature consistently showed discriminative power across all series, avoiding features that appeared important only at isolated or inconsistent time points. In this way, we ensured temporal consistency in feature relevance, which is crucial for reliable classification.

The following time points were selected from the 1st to the 169th based on their discriminative performance:

• Session 30 min: [64th, 65th, 69th, 70th]
• Session 24 h: [104th, 105th, 106th, 107th]
• Session 48 h: [80th, 81st, 82nd, 99th, 100th]

In the second step, we then identified the most informative features within those selected time points. The corresponding selected features for each session were as follows:

• Session 30 min: {SD₍ᵢ₎[HUB(Pᵢ)], min₍ᵢ₎[HUB(Pᵢ)], max₍ᵢ₎[HUB(Pᵢ)], min₍ᵢ₎[AUTH(Pᵢ)], min₍ᵢ₎[InDEG(Pᵢ)], max₍ᵢ₎[InDEG(Pᵢ)], min₍ᵢ₎[InCLOSE(Pᵢ)], CPL, Diameter}
• Session 24 h: {SD₍ᵢ₎[HUB(Pᵢ)], max₍ᵢ₎[HUB(Pᵢ)], min₍ᵢ₎[AUTH(Pᵢ)], min₍ᵢ₎[InDEG(Pᵢ)], CPL}
• Session 48 h: {max₍ᵢ₎[HUB(Pᵢ)], min₍ᵢ₎[AUTH(Pᵢ)], min₍ᵢ₎[InDEG(Pᵢ)], CPL}

Interestingly, this approach highlighted the presence of features that are consistently relevant across all 3 sessions, as well as features that play an important role only at specific sessions. This fact confirms that the signal pattern carries a highly structured level of information, which can complement classical spike-based statistics such as firing rate, burst activity, and others.

### 2D feature visualization

To exclusively provide a 2-dimensional (2D) visualization of the selected features and examine their possible discriminative properties, we applied principal component analysis (PCA), an unsupervised exploratory technique commonly used to represent multivariate data in a lower-dimensional space while retaining most of their information content [50].

**Fig. 5.**
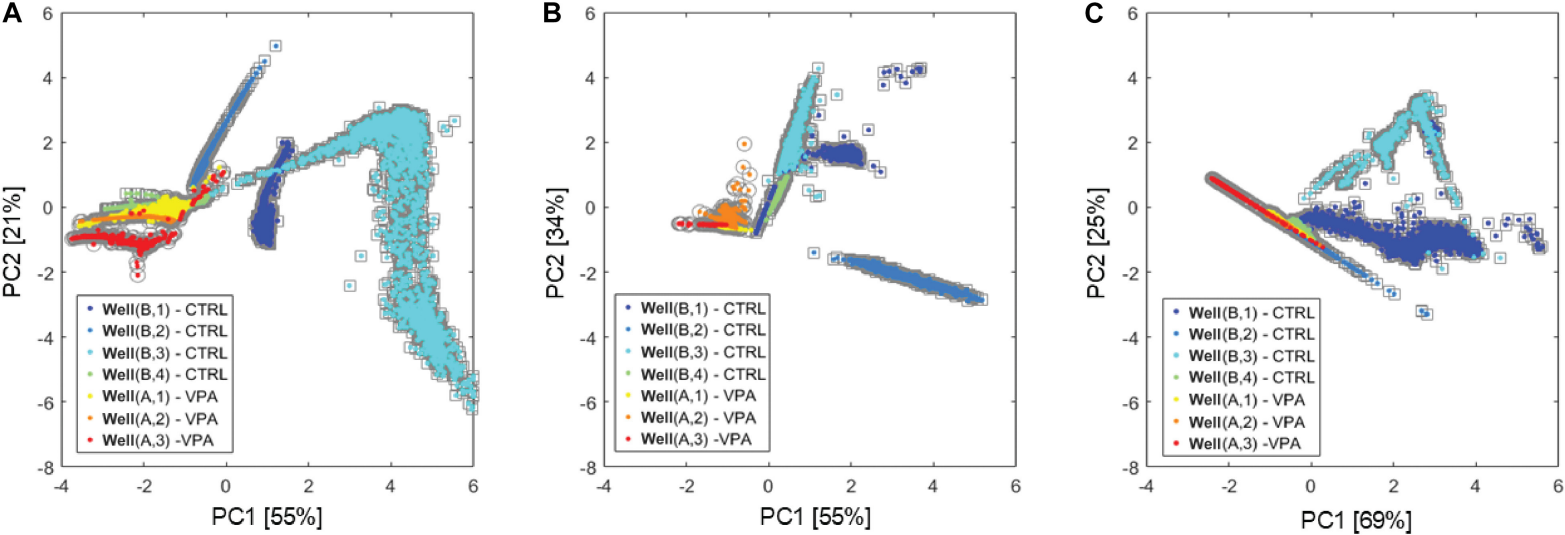
PCA results of graph descriptors for each session. (A to C) First and second principal components from features extracted for (A) session 30 min, (B) session 24 h, and (C) session 48 h. Percentage of variance explained is also indicated.

Fig. 5 shows the first 2 principal components of the normalized descriptors at 30 min, 24 h, and 48 h. Wells are color-coded from blue to cyan for the control (CTRL) condition and from yellow to red for the VPA-treated condition.

The PCA plots reveal that CTRL organoids (wells B1 to B4) exhibit a degree of inter-organoid heterogeneity, as reflected by the dispersion of points in the PCA space. In contrast, organoids exposed to VPA (wells A1 to A3) display reduced variability, suggesting a common effect induced by VPA that diminishes inter-organoid heterogeneity.

Moreover, this VPA-induced effect appears to evolve over time. The separation between CTRL and VPA-treated organoids becomes more pronounced from 30 min to 24 h post-exposure but tends to diminish by 48 h, indicating a time-dependent dynamic in the organoid response to VPA.

### Discrimination between CTRL and VPA exposure patterns

Building upon the class-separating trend observed in the PCA scores plot (Fig. 5), we employed the selected features to train a classification model aimed at predicting the effect of VPA.

Given the substantial heterogeneity in organoid behavior, even within the same experimental group, we adopted a LOWO approach. As discussed above, in this procedure, each of the 7 wells is left out in turn to serve as the test set, while the remaining 6 are used for training. This design provides a robust yet challenging framework to assess model generalization.

As an initial step, we assessed the ability of the classifier trained on the 30 min dataset (referred to as mdl_30min_) to discriminate between classes in subsequent recording sessions at 24 and 48 h.

**Fig. 6.**
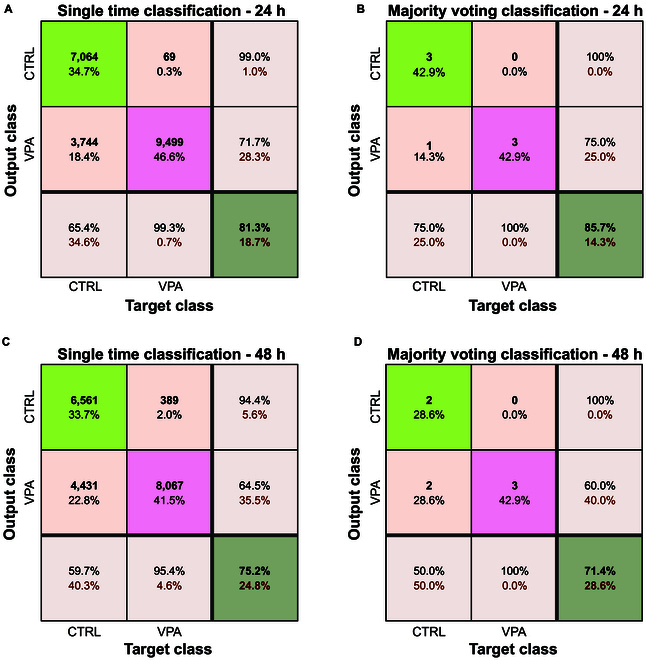
Classification results of the mdl_30min_ model when applied to 24 and 48 h. Two methodologies were exploited: classification in a single time point, labeling each time point independently as a CTRL or VPA, and well classification, where the final label for each of the 7 wells in total is assigned as the most frequent label across all time points of that well(Majority voting). (A) Single time point classification results when mdl_30min_ is tested on 24 h; (B) well classification through majority voting when mdl_30min_ is tested on 24 h; (C) single time point classification when mdl_30min_ is tested on 48 h; (D) well classification through majority voting when mdl_30min_ is tested on 48 h.

Interestingly, as the experimental time progresses, the distinction between the 2 classes becomes less pronounced (see Fig. 6).

Fig. 6A and C represent the results of single time point classification, where each time point is independently labeled when the model mdl_30min_ is tested on the dataset acquired at 24 and 48 h, respectively.

Fig. 6B and D represent the results of well classification, using majority voting, when the model mdl_30min_ is tested on the dataset acquired at 24 and 48 h, respectively, and the final label for each well is determined by the most frequent label across all the time points in the well. By 48 h, the model tends to mainly classify the samples as belonging to the VPA group, highlighting a convergence in activity patterns over time across all organoids. This result underscores the dynamic nature of the system, where both intrinsic organoid maturation and the VPA effect evolve over time.

These observations suggest 2 distinct sources of temporal variability: (a) the intrinsic developmental pathway of organoids; and (b) the time-dependent pharmacological impact of VPA. Therefore, to accurately monitor the effect of VPA, it is necessary to train separate models for each recording session, namely, mdl_30 min_, mdl_24 h_, and mdl_48 h_.

**Fig. 7.**
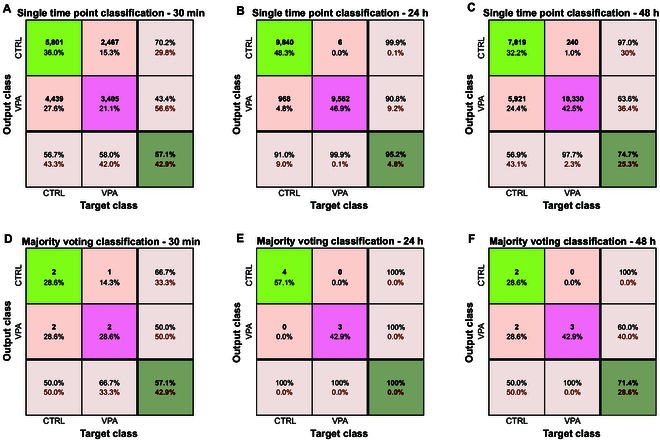
Classification results achieved for each session. (A and D) Thirty minutes, single time point and majority voting classification results, (B and E) 24 h, single time point and majority voting classification results, and (C and F) 48 h, single time point and majority voting classification results.

The results of the LOWO validation for these 3 models are presented in Fig. 7. Fig. 7A to C show the results of single time point classification, where each time point is independently labeled. In contrast, Fig. 7D to F present the outcomes after applying a majority voting strategy, in which the final label for each well is determined by the most frequent label across all the time points in a well.

To assess the robustness of the results, and after verifying data multicollinearity [51], we additionally tested 2 standard classification models: a support vector machine (SVM) with a radial basis function kernel (default settings) and a k-nearest neighbors (k-NN) classifier. For a fair comparison, the same cross-validation procedure and feature selection outcomes were applied. The corresponding accuracy values are reported in Table 4.

**Table 4.** Accuracy results obtained by applying the SVM method with radial basis function kernel and default setting and k-NN classification models.

	SVM with radial basis function (RBF) kernel					
	30 min		24 h		48 h	
	Single time	Majority voting	Single time	Majority voting	Single time	Voting majority
Accuracy	66%	71%	90%	100%	81%	88%
	KNN					
	30 min		24 h		48 h	
	Single time	Majority voting	Single time	Majority voting	Single time	Majority voting
Accuracy	64%	58%	93%	100%	66%	71%

Analysis of the confusion matrices and of the comparative results revealed several important findings. The highest classification accuracy occurs at 24 h, likely because the VPA effect becomes distinguishable, while the intrinsic activity of the organoids remains relatively stable. At 30 min, the pharmacological effect of VPA is not yet pronounced, resulting in weaker class separation, as illustrated in the confusion matrices of Fig. 7A and D. Moreover, at 30 min, the organoids exhibit a marked heterogeneity (as anticipated by the moderate separation in the PCA plot in Fig. 5), which further contributes to variability in the recordings and in the resulting misclassification. Conversely, at 48 h, the impact of VPA appears to diminish in comparison to the dominant effect of organoid maturation.

### Complexity of spike activity

Although the promising results obtained at very short timescales using MEA signals suggest clear potential for multiscale analysis, it remains essential to determine whether the observed discrimination relies on the multivariate approach itself or if similar insights can be extracted from the spiking activity recorded by individual electrodes. Planar MEA systems, by design, capture only extracellular recordings. In complex biological environments such as brain organoids, these extracellular signals reflect a composite of neuronal activity, making interpretation more challenging. For instance, previous studies [52,53], conducted in the cat primary visual cortex, have identified 5 distinct spike types from MEA recordings: regular-spiking, fast-spiking, triphasic-spiking, compound-spiking, and positive-spiking.

**Fig. 8.**
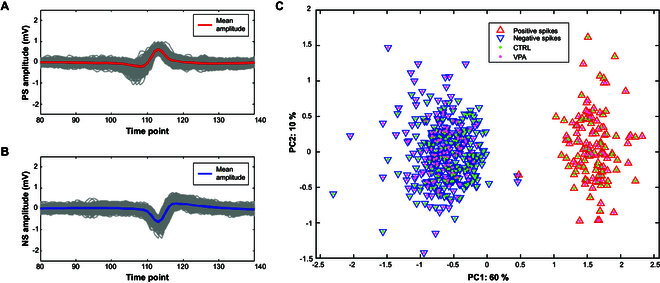
Spike classification. (A and B) Positive and negative spikes detected after k-means clustering. (C) PCA representation of positive (red) and negative spikes (blue). Green dots represent CTRL and pink dots represent VPA.

Motivated by these findings, we investigated whether different categories of spikes could also be identified in our dataset. Specifically, we applied an unsupervised clustering algorithm (k-means [54]) to the AM-spike signals recorded at the 24-h session that previously showed the highest discrimination between CTRL and VPA groups. The resulting clusters are shown in Fig. 8C, which presents the PCA score plot of the first 2 principal components derived from all spike waveforms, regardless of their electrode of origin and segment of acquisition.

Two key observations emerge from this analysis (Fig. 8C). First, k-means clustering revealed only 2 distinct spike classes, particularly positive and negative spikes, as shown in Fig. 8A and B. Second, when spike waveforms were labeled according to the treatment of their respective organoid (CTRL or VPA), no clear separation emerged between the groups in both spike classes. This strongly suggests that individual spike shapes alone are insufficient for distinguishing between experimental conditions.

To further test this hypothesis, we trained both linear (LDA) and nonlinear (SVM) classifiers using the spike waveforms from the 24-h session, again employing a LOWO cross-validation strategy. We conducted this analysis in 2 scenarios: first, using all spikes regardless of type, and then separately using only positive or negative spikes. In all cases, the classification accuracy remained below 50%, confirming that single spike signals, whether pooled or separated, do not carry sufficient discriminatory information on their own (see Table 5).

**Table 5.** LOWO classification results using the original AM-spikes acquired despite the electrode of origin or segment of acquisition.

Input spikes	Classification model	LOWO classification rate (%)
Positive and negative	LDA	40%
Positive	SVM	34%
Negative	LDA	43%

### Complementary standard brain activity descriptors

The final analysis on the spike data collected across the 3 experimental sessions focuses on standard spiking features commonly used to globally characterize neural activity. These features include the mean firing rate (MFR), the burst rate (BR), and the average number of spikes in a burst (SIB). These metrics were computed using the publicly available tool MEA-NAP [6] and were normalized to the average value recorded at baseline (0 h). To assess the temporal stability of these measures, we computed them using progressively increasing time windows—from 20 s up to 300 s, in 20-s increments. We expected each metric to stabilize after a certain duration of signal acquisition, reflecting the convergence of spiking activity to a steady-state behavior.

**Fig. 9.**
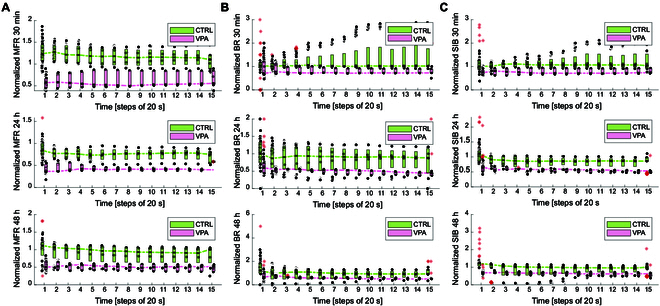
Standard spike and burst parameters. (A) Normalized MFR acquired at (top to bottom) 30 min, 24 h, and 48 h. (B) Normalized BR acquired at (top to bottom) 30 min, 24 h, and 48 h. (C) Normalized SIB acquired at (top to bottom) 30 min, 24 h, and 48 h. Orange boxes refer to VPA condition and blue boxes refer to CTRL. Red dots are for the outliers.

Fig. 9 presents the distributions of the 3 features, grouped in 20-s boxplots for the 7 analyzed wells. Fig. 9A shows the normalized MFR across the 3 sessions (30 min, 24 h, and 48 h, top to bottom), with orange boxes representing the VPA group and blue boxes denoting the CTRL. A clear separation is observed between the 2 conditions, supporting the findings from our earlier analysis. Notably, MFR values stabilize after approximately 1 min. As expected, VPA-treated organoids exhibit reduced firing rates, which aligns with the overall decrease in neuronal activity observed in the graph-based analysis.

Fig. 9B displays the BR across sessions, showing a similar trend to MFR: VPA exposure leads to a marked reduction in BR. At the 48-h session, an initial overlap between the 2 groups is seen, but this distinction becomes statistically significant as the recording window increases. Stability is achieved after about 2 min. Finally, Fig. 9C illustrates the distribution of the SIB feature. Several outliers are present in the control group, echoing the variability observed in earlier analyses (e.g., Fig. 5), where untreated organoids showed greater intrinsic heterogeneity. This variability tends to diminish in the VPA group, suggesting that VPA exposure may induce a more homogeneous neural response, which could be a relevant marker of altered or impaired functionality in the context of disease modeling.

## Conclusion 

Neuronal communication develops naturally as a network of interactions, in which the activity of local neuronal populations and their functional relationships can be represented as connections between nodes. Graph theory thus offers a principled framework to describe and quantify the complex, time-varying architecture of organoid activity.

For this purpose, we present a novel graph-based method for analyzing the electrical activity of neural organoids at millisecond resolution, providing a new lens through which to investigate the dynamic behavior of complex neuronal systems. By leveraging GDNs, our approach captures subtle and transient changes in network connectivity that are often overlooked by conventional electrophysiological metrics.

To validate the method, we analyzed 7 organoids recorded with a 16-electrode MEA under CTRL and VPA exposure, across 4 time sessions (0 h, 30 min, 24 h, and 48 h). Statistically significant alterations in descriptors such as characteristic path length, input degree, authority rank, and hub rank revealed a consistent decline in functional connectivity, most pronounced at 24 h. Classification models corroborated this finding, achieving their highest performance during the 24-h session.

Despite these promising findings, 2 main limitations should be acknowledged. First, the analyses were restricted to male-derived organoids, and the reported alterations may therefore primarily reflect male-specific responses. Second, the simplified forebrain organoid model employed here lacks microglia and other nonneuronal cell types and does not fully recapitulate the 6-layered structure of the human cortex. On the other hand, this simpler system allows us to directly evaluate the effect of VPA on neurons and more easily identify its impact on spiking signals and, subsequently, on genetic analysis. Future investigations including female-derived organoids and more complex forebrain organoid models will be essential to determine whether the observed graph-descriptor-based changes generalize across sexes and to clarify the role of astrocytes and microglia in the drug effect.

Building on this, we sought to interpret the biological significance of the selected descriptors (see Fig. 4). Node-level measures such as hub rank, authority rank, and indegree highlight the presence of highly connected or influential nodes, which may correspond to local “driver” regions of coordinated neuronal activity. Variations in these descriptors following VPA exposure suggest a disruption in the ability of specific regions to maintain central roles within the network, potentially reflecting a loss of synaptic integration capacity. Global descriptors such as characteristic path length and graph diameter instead capture the overall efficiency and spatial extent of communication: their increase under VPA indicates a shift toward less efficient and more fragmented signal propagation. Finally, measures of betweenness centrality, particularly their variability across nodes, provide an index of how balanced communication is across alternative pathways; changes in these values may signal the emergence of bottlenecks or the weakening of redundant communication routes.

Although a systematic biological validation remains beyond the scope of this study, these associations suggest that VPA exposure induces a topological reorganization of the network, consistent with the degradation of long-range functional connectivity and impaired cortical-like communication patterns. Importantly, our ability to monitor graph descriptors at the millisecond scale proved essential for detecting short-time variations in spike activity and transmission between connected neurons, insights that traditional global measures (e.g., MFR, BR, and average burst size) could not fully capture. While standard metrics confirmed a general trend toward more homogeneous yet less synergistic activity, our high-resolution graph-based approach also revealed the shaded reorganization of synaptic connectivity.

Taken together, these results demonstrate that graph descriptors derived from GDNs provide a powerful, noninvasive means of characterizing the functional architecture of neuronal networks with unprecedented temporal precision. Crucially, our method enables the identification of abnormalities using only a few milliseconds of MEA data across all electrodes, underscoring that extracellular spike signals contain rich, underutilized information about the health and dynamics of neural tissues.

Another key consideration is the generalizability of the GDN approach. The GDN was trained to forecast time-varying MEA recordings from individual human forebrain organoids, either untreated or exposed to VPA. The extracted graph features, reflecting connectivity strength and inferred causal interactions between electrodes, were then used for a separate inter-organoid classification task, aggregating descriptors across organoids by session. This separation of tasks mitigates the risk of overfitting at the classification level, although intra-organoid variability was carefully controlled through repeated cross-validation and balanced training/test folds when computationally feasible. Nonetheless, validation on independent datasets (e.g., different iPSC lines or labs) would further strengthen generalizability and represents an important direction for future research.

In addition, the temporal variability of discriminative features provides insight into both intrinsic development and drug-specific effects. Aging-related changes in control organoids manifested as increasing characteristic path length and reduced hub/authority ranks over time, capturing network maturation and synaptic weakening. In contrast, VPA-specific disruptions were most evident at 24 h, with reduced network efficiency and altered hub ranks, followed by partial reorganization at 48 h that may indicate compensatory mechanisms rather than full recovery. These observations highlight the value of graph descriptors not only for prediction but also for mechanistic interpretation of organoid network dynamics.

Finally, the proposed framework provides a precise spatiotemporal map of electrode activity, enabling the study of active versus inactive regions over time and laying the groundwork for future experiments involving active stimulation and dynamic response tracking. At present, such capabilities typically require invasive and expensive technologies like optogenetics or complex multimodal setups combining optics and transparent electronics [55,56]. Our approach thus opens new possibilities for noninvasive, high-resolution functional analysis of organoid networks and their perturbations.

## Data Availability

All the data are available upon request on the corresponding author.
